# Template‐Assisted Formation of High‐Quality α‐Phase HC(NH_2_)_2_PbI_3_ Perovskite Solar Cells

**DOI:** 10.1002/advs.201901591

**Published:** 2019-09-10

**Authors:** Pengju Shi, Yong Ding, Yingke Ren, Xiaoqiang Shi, Zulqarnain Arain, Cheng Liu, Xuepeng Liu, Molang Cai, Guozhong Cao, Mohammad Khaja Nazeeruddin, Songyuan Dai

**Affiliations:** ^1^ State Key Laboratory of Alternate Electrical Power System with Renewable Energy Sources North China Electric Power University Beijing 102206 China; ^2^ Beijing Key Laboratory of Novel Thin‐Film Solar Cells Beijing Key Laboratory of Energy Safety and Clean Utilization North China Electric Power University Beijing 102206 China; ^3^ Institute of Materials Science & Engineering University of Washington Seattle 98195 USA; ^4^ Group for Molecular Engineering of Functional Materials Institute of Chemical Sciences and Engineering École Polytechnique Fédérale de Lausanne (EPFL) CH‐1951 Sion Switzerland

**Keywords:** high quality, highest efficiency, perovskite solar cells, pure phase, template‐assisted

## Abstract

Formamidinium (FA) lead halide (α‐FAPbI_3_) perovskites are promising materials for photovoltaic applications because of their excellent light harvesting capability (absorption edge 840 nm) and long carrier diffusion length. However, it is extremely difficult to prepare a pure α‐FAPbI_3_ phase because of its easy transformation into a nondesirable δ‐FAPbI_3_ phase. In the present study, a “perovskite” template (MAPbI_3_‐FAI‐PbI_2_‐DMSO) structure is used to avoid and suppress the formation of δ‐FAPbI_3_ phases. The perovskite structure is formed via postdeposition involving the treatment of colloidal MAI‐PbI_2_‐DMSO film with FAI before annealing. In situ X‐ray diffraction in vacuum shows no detectable δ‐FAPbI_3_ phase during the whole synthesis process when the sample is annealed from 100 to 180 °C. This method is found to reduce defects at grain boundaries and enhance the film quality as determined by means of photoluminescence mapping and Kelvin probe force microscopy. The perovskite solar cells (PSCs) fabricated by this method demonstrate a much‐enhanced short‐circuit current density (  *J*_sc_) of 24.99 mA cm^−2^ and a power conversion efficiency (PCE) of 21.24%, which is the highest efficiency reported for pure FAPbI_3_, with great stability under 800 h of thermal ageing and 500 h of light soaking in nitrogen.

## Graphical abstract

**In this work, a “perovskite” template‐assisted structure** is developed to fabricate high‐quality α‐FAPbI_3_. The δ‐FAPbI_3_ phases are avoided. Defects are substantially reduced with an excellent light harvesting. A power conversion efficiency of 21.24% (the highest efficiency reported for pure α‐FAPbI_3_) is achieved. It also realizes a great stability in 800 h thermal ageing and 500 h light soaking.

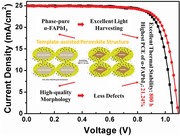

## 1 Introduction

Hybrid organic–inorganic perovskite solar cells (PSCs) have been distinguished as a potentially inexpensive and highly efficient technology with power conversion efficiencies (PCE) over 24.2%.^1^ Formamidinium‐based perovskites have attracted increasing attention owing to their good thermal stability, extended absorption range (≈840 nm), and long carrier diffusion lengths (hole/electron diffusion lengths = ≈813/≈177 nm).^2^, ^3^, ^4^, ^5^ However, it is extremely difficult to obtain phase‐pure and high‐quality FAPbI_3_ perovskite films compared with MAPbI_3_, mostly due to the ionic radius of the FA^+^ cation (2.53 Å), which is larger than that of the MA^+^ cation (2.17 Å). The larger cation leads to a high tolerance factor of FAPbI_3_ close to 1,^6^, ^7^, ^8^ indicating that the diffusion of the FA^+^ cation is difficult and that this perovskite structure is less stable than perovskite structures with the MA^+^ cation.^4^, ^9^ In addition, FAPbI_3_ is readily crystallized or transformed into an undesired nonperovskite byproduct (δ‐FAPbI_3_) during crystallization, which restricts the formation of pure cubic α‐FAPbI_3_ perovskite.^3^, ^10^, ^11^, ^12^, ^13^, ^14^ Thus far, developing and modifying the fabrication method of FAPbI_3_ perovskite films remain a major challenge in exploiting the advantages of α‐FAPbI_3_ in PSCs.

Hybrid perovskite thin films are typically derived from reactions between the organic and inorganic halide precursors through one‐step or two‐step processes.^15^, ^16^, ^17^ Many efforts have been reported to explore the formation and deposition of phase‐pure FAPbI_3_ perovskite thin films. Snaith and coworkers reported an effective method that involved adding a certain amount of hydroiodic acid (HI) into a perovskite precursor solution to fabricate α‐FAPbI_3_, but it failed to generate a uniform morphology.^2^ Zhao and coworkers introduced HPbI_3_ into perovskite precursors to prepare FAPbI_3_ films in a one‐step spin‐coating process.^18^ This method produced a relatively better surface than perovskite films fabricated from FAI/PbI_2_ and FAI/PbI_2_+HI precursor combinations, but the morphology needs further improvement for a good PSC. Although phase‐pure α‐FAPbI_3_ was obtained, the poor morphology had many defects and inferior long‐wavelength light conversion, which was demonstrated in the wavelength‐dependent external quantum efficiency (EQE).^14^, ^19^

In contrast, MAPbI_3_ perovskite thin films do not suffer from these challenges; it is easy to fabricate pure α‐MAPbI_3_ without the parasitic δ‐phase.^7^, ^20^ The significant processing advantages of MAPbI_3_ should be used to guide the fabrication of α‐FAPbI_3_. MAPbI_3_ might be converted directly into FAPbI_3_ perovskite with both the desirable morphologies and lattice structure of the original thin film preserved. To carry out this process, protonated FA^+^ cations must be dissolved in an alcohol solvent that also dissolves MAPbI_3_, affecting and destroying the original film morphology.^21^ Hence, it is necessary to modify the fabrication process for the preparation of α‐FAPbI_3_ with a compositional approach utilizing the 3D MAPbI_3_ structure.

In this work, with the template MAPbI_3_, a template‐assisted perovskite structure (MAPbI_3_‐(FAI‐PbI_2_‐DMSO) compound) was formed by a postprocessing method to produce pure‐phase α‐FAPbI_3_ with high‐quality morphology. The formation of δ‐FAPbI_3_ and the transformation of α‐MAPbI_3_ to α‐FAPbI_3_ were verified by variable temperature X‐ray diffraction (XRD), as there was no δ‐FAPbI_3_ fingerprint peak during the annealing process from 100 up to 180 °C. This method involved dripping a certain amount of FAI/IPA solution on the top of colloid‐like MAI‐PbI_2_‐DMSO films, and a template‐assisted perovskite structure was formed during annealing at 100 °C. After annealing was performed at 140 °C for 60 min, purified α‐FAPbI_3_ was obtained. Meanwhile, as determined by mapping measurements, the template structure achieved high‐quality morphology and a substantially reduced number of deep defects. It is reported that deep trap states mainly stemming from the surface would enhance the risk of recombination and lead to inferior quantum efficiency at the high‐wavelength range.^14^, ^19^, ^22^ Consequently, this method can not only fabricate pure α‐FAPbI_3_ but also achieve a higher voltage. The FAPbI_3_‐based PSC achieved a PCE of 21.24% with high stability under 800 h of thermal ageing and 500 h of light soaking with encapsulation.

## 2 Results and Discussions

**Figure 1:**
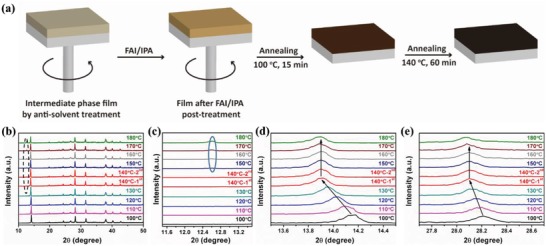
a) Schematic illustration of postprocessing for the fabrication of FA‐based perovskite films. b–e) In situ X‐ray diffraction of the perovskite films in vacuum during postprocessing. The temperature increases at 5 °C min^−1^, and the soaking time of each scan is 10 min with a characterization speed of 10° per min.

**Figure** **1**a schematically illustrates the fabrication process for the preparation of FA‐based perovskite films. A perovskite precursor solution (MAI:PbI_2_ = 1:1) was spin‐coated on mesoporous TiO_2_/FTO substrate following a reported procedure.^17^ After chlorobenzene (CB) was dripped, the FAI/isopropanol (IPA) solution was quickly dripped onto the spinning unannealed colloidal film, and the color changed to maple. When the sample was annealed at the initial temperature of 100 °C, the color (maple) transformed into dark brown, and the final film turned completely black after the second stage of annealing, which occurred at 140 °C for an hour. Figure 1b–d shows the in situ X‐ray diffraction patterns at various temperatures in vacuum. During the annealing from 100 to 180 °C, the absence of a characteristic peak of δ‐FAPbI_3_ (11.8°) (Figure 1c) indicated that there is no formation of δ‐FAPbI_3._ The peaks corresponding to the (110) and (220) planes showed a shift in position from ≈14.2° to 13.9° and from ≈28.2° to ≈28.1°, respectively.^4^, ^16^, ^18^, ^23^ Such peak position shifts strongly suggested that the α‐MAPbI_3_ was transformed to α‐FAPbI_3_ without the formation of the undesired δ‐FAPbI_3_ phase. In contrast, the in situ XRD patterns of the perovskite films prepared by the conventional method revealed a peak of δ‐FAPbI_3_ (11.8°) (Figure S2, Supporting Information). The formation of δ‐FAPbI_3_ would influence the growth of α‐FAPbI_3_ perovskite during annealing at 140 °C, and a part of δ‐FAPbI_3_ may transform into α‐FAPbI_3_ (13.9° and 28.1°). The main δ‐FAPbI_3_ probably decomposes to PbI_2_.^13^, ^14^

**Figure 2:**
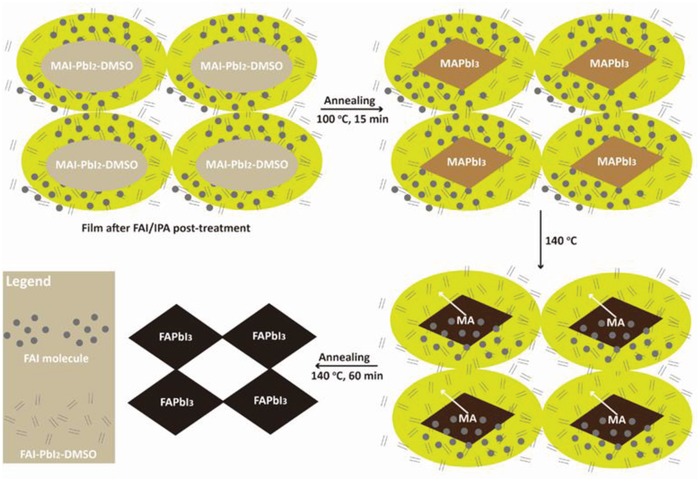
Schematic illustration of the template‐assisted perovskite mechanism for the fabrication of phase‐pure α‐FAPbI_3_ perovskite films.

**Figure** **2** schematically illustrates our proposed mechanism of the template‐assisted perovskite formation. First, the perovskite precursor solution (MAI+PbI_2_+DMSO) was spin‐coated onto the substrate. The MAI+PbI_2_+DMSO films transformed into 2D MAI‐PbI_2_‐DMSO (Figure S3, Supporting Information) when CB was dripped.^17^ After FAI solution was dripped, the colloidal MAI‐PbI_2_‐DMSO converted into the intermediate of (MAI‐PbI_2_‐DMSO)+(FAI‐PbI_2_‐DMSO), which resulted from the FAI diffusing into a 2D structure, and the MAI molecules of the outer shell of MAI‐PbI_2_‐DMSO could be easily substituted by additional FAI molecules with the inner MAI‐PbI_2_‐DMSO structure remaining unaltered. During the initial annealing at 100 °C, the perovskite template‐assisted structure MAPbI_3_‐FAI‐PbI_2_‐DMSO was formed because the remaining MAI‐PbI_2_‐DMSO inner structure transformed to MAPbI_3_ and coordinated with the outer FAI‐PbI_2_‐DMSO.^24^, ^25^ When annealed at 140 °C, some of the FA^+^ cations remaining in the film from the dripping FAI/IPA solution, intercalated into the MAPbI_3_ lattice to substitute for MA^+^ as a result of the reduction of MA^+^ to MA and the oxidation of FA to FA^+^ at higher temperature.^6^ In the meantime, the coordinated FAI‐PbI_2_‐DMSO could convert to FAPbI_3_ on the template of the 3D MAPbI_3_ structure. After one hour of annealing, the purified FAPbI_3_ was finally obtained without the formation of a δ‐FAPbI_3_ phase during the whole process. The step‐wise compositional process of the template‐assisted perovskite structure can be described as^16^, ^26^

(1) $$\begin{array}{l} \mathrm{MAI}+{\mathrm{PbI}}_{2}+\mathrm{DMSO}\overset{CB}{\to }\mathrm{MAI}-{\mathrm{PbI}}_{2}-\mathrm{DMSO}\overset{FAI}{\to }x\left(\mathrm{FAI}-{\mathrm{PbI}}_{2}-\mathrm{DMSO}\right)\\ +\left(1-x\right)\left(\mathrm{MAI}-{\mathrm{PbI}}_{2}-\mathrm{DMSO}\right)+\left(1-x\right)\mathrm{FAI}+x\mathrm{MAI}\overset{100\;\;{}^{\circ}C}{\to }\\ \left(1-x\right){\mathrm{MAPbI}}_{3}-x\left(\mathrm{FAI}-{\mathrm{PbI}}_{2}-\mathrm{DMSO}\right)+\left(1-x\right)\mathrm{FAI}+\left(1-x\right)\mathrm{DMSO}↑\\ +x\mathrm{MAI}\overset{140\;\;{}^{\circ}C}{\to }{\mathrm{FAPbI}}_{3}+\mathrm{MAI}↑+x\mathrm{DMSO}↑ \end{array}$$

To verify the intermediate phase of MAPbI_3_‐FAI‐PbI_2_‐DMSO, XRD patterns of the corresponding intermediate products were measured, and they are shown in Figure S4 in the Supporting Information. The lack of the characteristic peak of δ‐FAPbI_3_ (11.8°) indicates that δ‐FAPbI_3_ does not exist throughout the whole process. As shown in Figure S4a in the Supporting Information, the characteristic peaks of MAI‐PbI_2_‐DMSO (Figure S4b, Supporting Information) (marked with “*”) and FAI‐PbI_2_‐DMSO (Figure S4c, Supporting Information) (marked with “#”) appeared in the intermediate product after FAI dripping. There are two small peaks at 6.54° and 7.20° (Figure S4a, Supporting Information) in the intermediate product, which represent a small quantity of MAI‐PbI_2_‐DMSO.^25^ The presence of those peaks indicates the formation of MAI‐PbI_2_‐DMSO+FAI‐PbI_2_‐DMSO in the interim, which was also proven by the FTIR spectra shown in Figure S5 in the Supporting Information. The C=N stretch (1712 cm^−1^) indicated the existence of FA^+^ in the intermediate phase, confirming the formation of MAI‐PbI_2_‐DMSO+FAI‐PbI_2_‐DMSO.^15^, ^27^

After the samples were annealed at 100 °C, the characteristic peaks of MAPbI_3_ (marked with “&”) and the FAI‐PbI_2_‐DMSO intermediate appeared in the template‐assisted perovskite structure (Figure S4e, Supporting Information), indicating a mixture of 2D/3D structures.^14^, ^28^ However, the peak of FAI‐PbI_2_‐DMSO at 24.3° (002) (Figure S4c, Supporting Information) and the peak of MAPbI_3_ at 14.08° (110) (Figure S4f, Supporting Information) disappeared when the samples were annealed at 100 °C.^7^ This result may indicate that the intermediate of FAI‐PbI_2_‐DMSO and MAI‐PbI_2_‐DMSO transformed into a template 2D/3D perovskite structure that is not a δ‐phase but is advantageous for the production of the phase‐pure α‐FAPbI_3_. The formation of MAPbI_3_‐FAI‐PbI_2_‐DMSO may derive from the connection between the (002) plane of FAI‐PbI_2_‐DMSO and the (110) plane of MAPbI_3_, which was evidenced by the disappearance of the XRD peaks of FAI‐PbI_2_‐DMSO and the appearance of new peaks of MAPbI_3_. The peaks at 1712 and 600 cm^−1^ (NH_2_) in the Fourier transform infrared (FTIR) spectra in Figure S6 in the Supporting Information indicated the presence of FA.^27^, ^29^ In addition, the CH_3_‐NH_3_^+^ rock (991 cm^−1^) and stretch (943 and 961 cm^−1^) demonstrated the formation of α‐MAPbI_3_.^30^ Therefore, it can be concluded that the template‐assisted perovskite structure is MAPbI_3_‐FAI‐PbI_2_‐DMSO.

During the annealing to 140 °C, FA^+^ substituted for MA^+^ (in the MAPbI_3_ lattice), and the template‐assisted perovskite structure transformed to FAPbI_3_ with the help of MAPbI_3_. Finally, phase‐pure FAPbI_3_ perovskite was obtained. Moreover, the amount of MAI‐PbI_2_‐DMSO converted depended on the quantity of FAI dripped because the more FAI cations that diffuse to the film, the more MAI will be substituted, up to the limit of the MAI concentration. The FAI concentration could directly influence the effectiveness of the template‐assisted perovskite structure because it would increase its reactivity with MAPbI_3_ in the center of each particle, which would then be transformed to phase‐pure FAPbI_3_. However, a low number of FAI molecules would result in excess PbI_2_ in the film and the formation of many defects.^31^ Proper FAI concentration is critical for the formation of phase‐pure and high‐quality FAPbI_3_ perovskite films.

**Figure 3:**
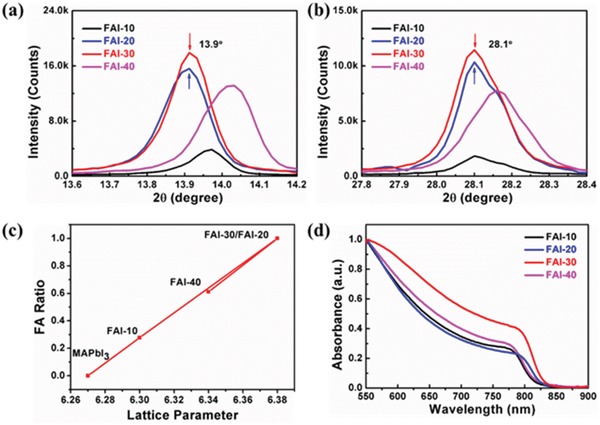
XRD peaks for the a) (110) plane and b) (220) plane of the films postprocessed with different FAI concentrations (10, 20, 30, and 40 mg mL^−1^). c) The lattice parameter of the postprocessed films with different FA contents. The lattice parameter is calculated from the Bragg equation: 2*d*sin*θ = nλ* (λ = 1.54056 Å). The line is a linear fitting of the lattice parameters. d) UV–visible absorption spectra of the postprocessed films.

To further study the influence of the FAI postprocessing on the lattice structure and phase purity of the final perovskite film, films were postprocessed with different FAI concentrations in solution (10, 20, 30, and 40 mg mL^−1^), and the samples were named FAI‐10, FAI‐20, FAI‐30, and FAI‐40, respectively. As shown in **Figure** **3**a–b and Figure S7 in the Supporting Information, the XRD peaks of (110) and (220) facets appeared at 13.9° and 28.1°, respectively, for both FAI‐20 and FAI‐30.^3^, ^11^ However, when processed for FAI‐10 and FAI‐40, the peaks of the (110) plane and (220) plane were close to 13.9° and 28.1°, respectively, demonstrating that there was significant FA content and that the phase was not pure FAPbI_3_. It is evident in Figure 3c that the lattice parameter of FAI‐20 and FAI‐30 is 6.38 Å, which verifies the formation of the α‐FAPbI_3_ lattice.^12^ To further certify the purity of the α‐FAPbI_3_ perovskite films, nuclear magnetic resonance (NMR) with low‐temperature ^13^C cross‐polarized magic angle spinning (CP MAS) spectra were measured. Figure S8 in the Supporting Information shows the peaks of MAPbI_3_ and FAPbI_3_ from the conventional method (C‐MAPbI_3_ and C‐FAPbI_3_) at ≈31 ppm (Figure S8a, Supporting Information) and ≈156 ppm (Figure S8b, Supporting Information), respectively.^32^ As shown in Figure S8a,b in the Supporting Information, there was only an NMR peak at ≈156 ppm in FAI‐20 and FAI‐30, which verifies an absence of δ‐FAPbI_3_ and MA^+^ in the films. However, both FAI‐10 and FAI‐40 have NMR peaks at ≈156 and ≈31 ppm, respectively. Meanwhile, the corresponding ^1^H NMR spectra are shown in Figure S8c in the Supporting Information.^33^ These spectra indicated the same results as the ^13^C spectra. These results proved that both FAI‐30 and FAI‐20 were completely purified α‐FAPbI_3_ without δ‐phase, while FAI‐10 and FAI‐40 turned into a mixed cation perovskite with high FA content. All of the results are in agreement with the XRD measurements, and together they support the production of phase‐pure α‐FAPbI_3_ by the template‐assisted perovskite technique. In addition, C‐FAPbI_3_ also exhibits δ‐FAPbI_3_ (Figure S9a, Supporting Information). As a result, it is difficult to obtain phase‐pure FAPbI_3_ when treatment uses 10 and 40 mg mL^−1^ of FAI, which is consistent with the earlier mentioned consideration that less or more FAI than 20–30 mg mL^−1^ would restrict the formation of phase‐pure FAPbI_3_.

Figure 3d shows the UV–visible absorption spectra of postprocessed films formed with different FAI concentrations. The FAI‐30 film shows a broad absorption edge at 840 nm (pure FAPbI_3_), but the FAI‐20 film displays a weaker absorption, which owes to the excess residual PbI_2_ content in the film.^31^, ^34^ As a comparison, the C‐FAPbI_3_ film shows a blue shift of the absorption edge (Figure S9b, Supporting Information). In addition, the excess PbI_2_ would restrict the growth of the grains and create a gap in the grain boundaries. Furthermore, the more FAI would allow a small concentration of MAI‐PbI_2_‐DMSO to remain in the film and affect the formation of the template‐assisted perovskite, which in turn would result in a poor morphology.^31^, ^35^ Therefore, it is essential to judiciously control the FAI content in the FAI solution to preserve the morphology of the unannealed film, which can lead to a good quality of pure‐phase FAPbI_3_ film.

**Figure 4:**
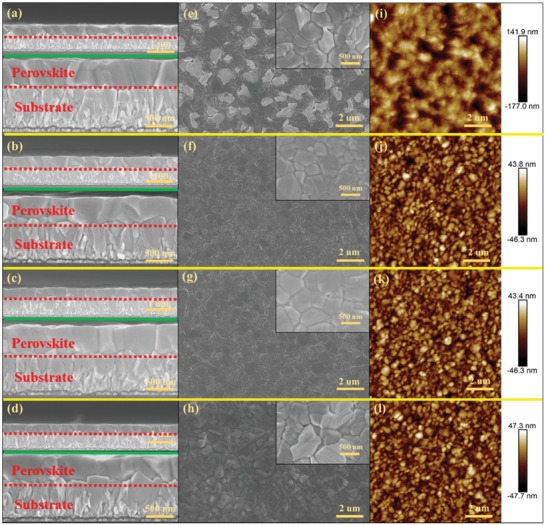
Cross‐sectional scanning electron microscopy (SEM) images: a) FAI‐10, b) FAI‐20, c) FAI‐30, and d) FAI‐40. The yellow line distinguishes the different samples. The green line distinguishes the cross‐sectional SEM at different microscope magnifications (the top is 2000×, while the below is 1000×). The red line distinguishes the perovskite and substrate. Corresponding top‐view SEM images of e) FAI‐10, f) FAI‐20, g) FAI‐30, and h) FAI‐40 films. Atomic force microscope (AFM) images of i) FAI‐10, j) FAI‐20, k) FAI‐30, and l) FAI‐40 films.

The morphological textures of the perovskite films are shown in **Figure** **4**. It was previously reported that morphologies with a large grain size, compactness, and a flattened surface is key to attaining high photovoltaic performance.^36^ The cross‐sectional scanning electron microscopy(SEM) images (Figure 4) show that the postprocessed films formed with different FAI concentrations are uniform and could be clearly identified through an integral large grain with visible boundaries in the vertical direction.^37^ No pinhole or visible defect on the grains was observed, and the estimated thickness of the absorbers is 400–500 nm, which are completely uniformly spread on the top of the electron transport layer and demonstrate a high‐quality perovskite film.^38^ The top‐view SEM images (Figure 4e–h) of perovskite layers show smooth, uniform and pinhole‐free polygonal grains with sizes of 400–500 nm. In contrast, the FAI‐10 film displays excess lead iodide (PbI_2_) remaining in the film, probably due to an insufficient number of FAI molecules for the reaction, which is also in line with the PbI_2_ peak (Figure S7, Supporting Information). The residual PbI_2_ affects the quality of the film morphology and restricts the formation of phase‐pure FAPbI_3_.^39^ The FAI‐30 film shows a smooth and flattened morphology in which grain size increases to up to ≈500 nm in size.^40^ As a control, C‐FAPbI_3_ has a rough surface with poor cross‐sectional properties (Figure S12, Supporting Information). The improved morphology could enhance light harvesting and increase the external quantum efficiency. The spectral response of the treated films is confirmed by the EQE spectrum in Figure S10 in the Supporting Information. As expected, the EQE spectra exhibited a redshift with increasing FAI concentration, and the maximum absorption edge was at a wavelength greater than 833 nm, which has been previously reported in the mixed cation perovskites FA*_x_*MA_1−_ *_x_*PbI_3_ with high FA content (*x* = 0.7 and 0.9).^14^, ^41^ This phenomenon could be due to several factors. First, FA*_x_*MA_1−_ *_x_*PbI_3_ (*x* = 0.28, 1.00, 1.00, and 0.63) is a good composition for enhanced PV performance and promises increased photoelectric conversion in the high wavelength range. Second, film fabrication via the postprocessing method is helpful for modifying different compositions of FA*_x_*MA_1−_ *_x_*PbI_3_ perovskites. Third, the electron/hole diffusion length (≈177/≈813 nm) of the FAPbI_3_‐based device is suitable for FTO/Cl‐TiO_2_/meso‐TiO_2_/perovskite/spiro structures.^3^ As shown in Figure 4i–l, both the FAI‐20 and FAI‐30 films have a smooth surface and a roughness of −46–43 nm. Though the FAI‐40 film has a similar surface, its roughness is larger than that of the FAI‐20 and FAI‐30 films. The FAI‐10 film is coarse because excess PbI_2_ influences the morphology. For the different morphologies of purified FAPbI_3_ (Figure 4e–h), it is important to analyze the defects in the purified α‐FAPbI_3_ films caused by the different FAI concentrations.^42^

**Figure 5:**
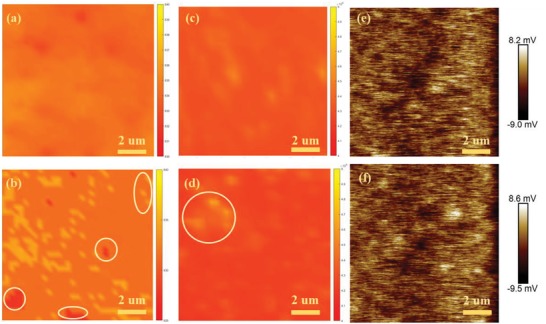
Confocal photoluminescence (PL) peak maps of the a) FAI‐30 and b) FAI‐20 configurations infiltrated into a nonquenching compact layer and measured through the glass side. The corresponding peak intensity maps of c) FAI‐30 and d) FAI‐20 films, respectively. Kelvin probe force microscope (KPFM) images of e) FAI‐30 and f) FAI‐20 films, respectively. All of the areas are 10 × 10 µm^2^ each.

To understand the quality of the purified FAPbI_3_ films, confocal photoluminescence (PL) mappings of FAI‐20 and FAI‐30 films were performed with a structure of glass/perovskite.^43^ **Figure** **5**a,b shows the confocal PL peak maps with excitation through the glass. The FAI‐30 film showed an extremely uniform emission distribution (830–840 nm) that proved the high quality and excellent uniformity of α‐FAPbI_3_ (Figure 5a).^20^ For the FAI‐20 maps in Figure 5b, the peaks ranged from 825 to 840 nm. This result suggested that the components of the film in the small circle were slightly nonuniform due to insufficient FAI to exchange with MAI and that the MAI participated in the lattice. The corresponding intensity maps were measured, and they are shown in Figure 5c,d to demonstrate the dynamic carrier kinetics. FAI‐30 showed the most uniform peak intensity and thus demonstrated minimal nonradiative decay; its intensity is almost an order of magnitude higher than that of the FAI‐20. In addition, there are light and dark spots in the FAI‐20 film, showing different peak intensities. The nonuniformity results in trap states and contributes to quality of FAI‐20 being poorer than that of FAI‐30.^44^ In contrast, PL maps of the C‐FAPbI_3_ film show extensive absorption (670–820 nm) and nonuniform intensity (Figure S13a,b, Supporting Information), similar to the atomic force microscope(AFM) image, which has a rough surface (Figure S13c, Supporting Information).

To determine the trap states and uniformity of the FAI‐20 and FAI‐30 films, Kelvin probe force microscopy (KPFM) was employed to measure the surface potential of the perovskite films, and the results are shown in Figure 5e,f. Different concentrations of FAI solution induce a change in the surface work function, which can be detected in the KPFM measurement.^43^, ^45^ Compared with the FAI‐20 film, the FAI‐30 film shows a uniform surface potential throughout the whole scanning region (Figure 5e), confirming the film uniformity.^46^ However, the C‐FAPbI_3_ film (Figure S13d, Supporting Information) failed to realize a superb uniformity. Good uniformity helps to improve contact between the hole transport layer (HTL) and perovskite layer, reducing the number of trap states and realizing a decreased barrier height. All of the above factors are significant for the transport of holes and, as a result, balancing charge transport.

**Figure 6:**
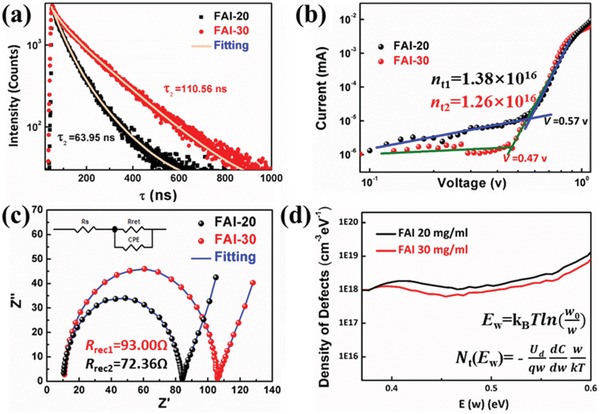
Analysis of defects on the FAI‐20 and FAI‐30 films via a) time‐resolved photoluminescence (TRPL) spectra, b) space‐charge‐limited current (SCLC) measurements, c) electrochemical impedance spectroscopy (EIS) and d) density of defect measurements.

To further investigate the trap states of the perovskite films and devices, defect measurements were conducted, and the results are shown in **Figure** **6**. Steady‐state photoluminescence and time‐resolved photoluminescence (TRPL) methods were used to study the trap states in the perovskite bulks.^47^ We prepared perovskite films on bare glass to avoid charge injection between the active layer and FTO. As shown in Figure S11a in the Supporting Information, the FAI‐30 film exhibits a sharper and stronger peak than the other films, indicating a reduction in defects on the surface of film. The charge lifetime (Figure 6a) was fitted by the double index model $\left(y\;=\;A_{1}\exp\left(-\frac{t}{\tau_{1}}\right)+A_{2}\exp\left(-\frac{t}{\tau_{2}}\right)\right)$, the effective time was calculated via $\tau_{eff}=\;\frac{A_{1}\tau_{1}+A_{2}\tau_{2}}{A_{1}+A_{2}}$,^48^ and the results are summarized in Table S1 in the Supporting Information. The average lifetime (τ_eff_) indicates a longer carrier lifetime (110.56 ns) and fewer defects for the FAI‐30 film than for the FAI‐20 film (63.95 ns), which was in line with the steady‐state PL results (Figure S11a, Supporting Information). To further investigate the electron–hole mobility of the postprocessed films, the space‐charge‐limited current (SCLC) was measured for an FTO/ETL/perovskite/Au structure. The defect density (*n*_t_) was calculated according to the following equation

(2) $$V_{\mathrm{TFL}}=\frac{en_{t}d^{2}}{2\varepsilon \varepsilon_{0}}$$

where *e* is the electric charge (1.602 × 10^−19^ C), *d* is the thickness of the perovskite layer, ε_0_ is the vacuum permittivity (8.8542 × 10^−14^ F cm^−1^), and ε is the dielectric constant.^49^, ^50^ Compared with the FAI‐20 film, the FAI‐30 film shows a reduced onset voltage for the trap‐filled limit (*V*_TFL_, Figure 6b), demonstrating a reduced defect density (*n*_t2_ < *n*_t1_). The open‐circuit voltage decay curves in Figure S11b in the Supporting Information display similar trends as the PL and TRPL results. The FAI‐30 film shows a slower decay than the FAI‐20 film. To study the interfacial transfer dynamics of devices, electrochemical impedance spectroscopy (EIS) data were fitted in Figure 6c, and they are summarized in Table S2 in the Supporting Information.^51^ The recombination resistance (*R*_rec_) of the FAI‐30 and FAI‐20 films was 93.00 and 72.36 Ω, respectively, suggesting that the capability of the films to suppress nonradiative recombination losses becomes stronger as the *R*_rec_ values increase. Figure 6d shows the calculated density of the defects, which were deep trap states, stemming from the surface of the perovskite layer.^50^, ^52^ It is suggested that the FAI‐30 film is superior because it has fewer defects than the other films, which is well demonstrated by previously discussed results. Figure S14 in the Supporting Information shows the quantity of defects in the C‐FAPbI_3_ film, indicating an increased number of trap states that is higher than the number in the FAI‐30 and FAI‐20 films. All of the above measurements proved that FAI treatment of films could substantially reduce grain boundaries and defects.^47^

**Figure 7:**
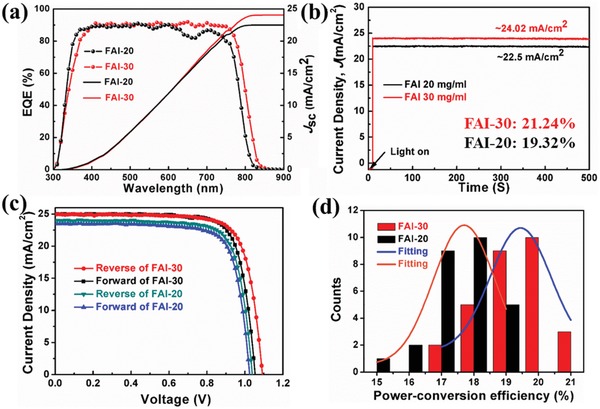
a) EQE spectra of the best‐performing devices based on FAI‐20 and FAI‐30 films with respective integrated *J*_sc_ curves. b) Stabilized *J*_sc_ at the maximum power point of FAI‐20‐ and FAI‐30‐based devices. c) *J–V* curves of the best FAI‐20‐ and FAI‐30‐based devices. d) Histogram of average efficiencies of FAI‐20‐ and FAI‐30‐based devices (28 devices).

The external quantum efficiency and PCE were measured to examine the total effects of the FAI postprocessing method on the performance of the final prepared devices. **Figure** **7**a exhibits substantially enhanced *J*_sc_ produced by increased effective carrier collection at both electrodes, and the value of the integrated current reached 24.02 mA cm^−2^, which is close to the measured *J*_sc_ of 24.99 mA cm^−2^. The stabilized *J*_sc_ at the maximum power point for the same device was measured over the time range of 0–500 s, showing that the current stabilized at ≈24.02 mA cm^−2^, which is consistent with the IPCE results. The current density–voltage (*J*–*V*) characteristics of FAI‐20‐ and FAI‐30‐based devices are shown in Figure 7c and listed in Table S3 in the Supporting Information. Compared with C‐FAPbI_3_ (Figure S15b, Table S3, Supporting Information), the best postprocessed device had a PCE of 21.24%, a *J*_SC_ of 24.99 mA cm^−2^, a *V*_OC_ of 1.09 V, and an FF of 78.01%. Moreover, there is little detected hysteresis in the reverse direction and the forward direction. In Figure 7d, a histogram of the average PCEs for FAI‐20 and FAI‐30 is presented. Compared with the cells of C‐FAPbI_3_ (Figure S16, Supporting Information), ≈80% of FAI‐30 cells exhibited an overall efficiency exceeding 19% at 1 sun.

Figure S17 in the Supporting Information shows the stability of an α‐FAPbI_3_‐based device aged under thermal and light soaking conditions (25 °C). The photovoltaic performance of FAPbI_3_ was measured every 24 h of ageing at room temperature (25 °C), 85, 140, and 195 °C.^24^ The FAI‐30‐based device showed a high thermal stability, which might be attributed to its high‐quality morphology and improved phase stability. In addition, the FAI‐30‐based devices showed ≈25% degradation after 500 h of light soaking. The stability of the devices tested in nitrogen showed substantial improvement over that of devices tested in air (Figure S18, Supporting Information). All of the above results reinforce the hypothesis that the fabrication of FAPbI_3_ perovskite films assisted by the formation of a template‐assisted perovskite structure could enhance the photoelectric performance because of the reduced number of defects reducing light and thermal degradation.

## 3 Conclusions

This template‐assisted approach enables the preparation of high‐quality and phase‐pure α‐FAPbI_3_ perovskite layers. The resulting α‐FAPbI_3_ film showed an absorption edge at a wavelength greater than 840 nm and highly uniform and enlarged grains (400–500 nm). The enhanced film quality increased the photon‐induced electron–hole pair mobility and effective light utilization. The improved crystallinity and reduced grain boundaries of the perovskite film effectively reduced the nonradiative carrier recombination. The devices assembled by employing the FAI postprocessing technique showed the highest power conversion efficiency (21.24%) under standard illumination. This study provides an effective protocol for fabricating efficient and stable inorganic–organic hybrid heterojunction solar cells, which can be helpful in the future for producing commercially feasible PSCs. This work has realized the highest reported efficiency of FAPbI_3_ with superb stability under 800 h of thermal ageing and 500 h of light soaking.

## Conflict of Interest

The authors declare no conflict of interest.

## Supporting information

Supplementary
