# Supplementary material for: Template‐Assisted Formation of High‐Quality α‐Phase HC(NH2)2PbI3 Perovskite Solar Cells
Source: Adv Sci (Weinh). 2019 Sep 10;6(21):1901591. doi: 10.1002/advs.201901591 (PMC6839747; doi:10.1002/advs.201901591)
Supplement: Supplementary file 1 — Supplementary [file ADVS-6-1901591-s001.pdf]

Copyright WILEY-VCH Verlag GmbH & Co. KGaA, 69469 Weinheim, Germany, 2019.

ADVANCED SCIENCE
Open Access

# Supporting Information

for *Adv. Sci.,* DOI: 10.1002/advs.201901591

# Template-Assisted Formation of High-Quality α-Phase $HC(NH_2)_2PbI_3$ Perovskite Solar Cells

*Pengju Shi, Yong Ding,* Yingke Ren, Xiaoqiang Shi, Zulqarnain Arain, Cheng Liu, Xuepeng Liu, Molang Cai,* Guozhong Cao, Mohammad Khaja Nazeeruddin,* and Songyuan Dai**

# Template-assisted Formation of High-quality α-phase $HC(NH_2)_2PbI_3$ Perovskite Solar Cells

*Pengju Shi*[a,b], *Yong Ding*[a,b,*], *Yingke Ren*[a,b], *Xiaoqiang Shi*[a,b], *Zulqarnain Arain*[a,b], *Cheng Liu*[a,b], *Xuepeng Liu*[b], *Molang Cai*[a,b*], *Guozhong Cao*[c], *Mohammad Khaja Nazeeruddin*[d,*] *and Songyuan Dai*[a,b,*]

[a]State Key Laboratory of Alternate Electrical Power System with Renewable Energy Sources, North China Electric Power University, Beijing, 102206, China.

[b]Beijing Key Laboratory of Novel Thin-Film Solar Cells, Beijing Key Laboratory of Energy Safety and Clean Utilization, North China Electric Power University, Beijing, 102206, China.

[c]Institute of Materials Science & Engineering, University of Washington, Seattle, 98195, United States.

[d]Group for Molecular Engineering of Functional Materials Institute of Chemical Sciences and Engineering École Polytechnique Fédérale de Lausanne (EPFL), CH-1951 Sion, Switzerland.

*Corresponding authors:

E-mail: dingy@ncepu.edu.cn (Dr. Y. Ding)

E-mail: molangcai@ncepu.edu.cn (Prof. M.L Cai)

E-mail: mdkhaja.nazeeruddin@epfl.ch (Prof. M. D. Nazeeruddin)

E-mail: sydai@ncepu.edu.cn (Prof. S. Y. Dai)

**Supporting Information**

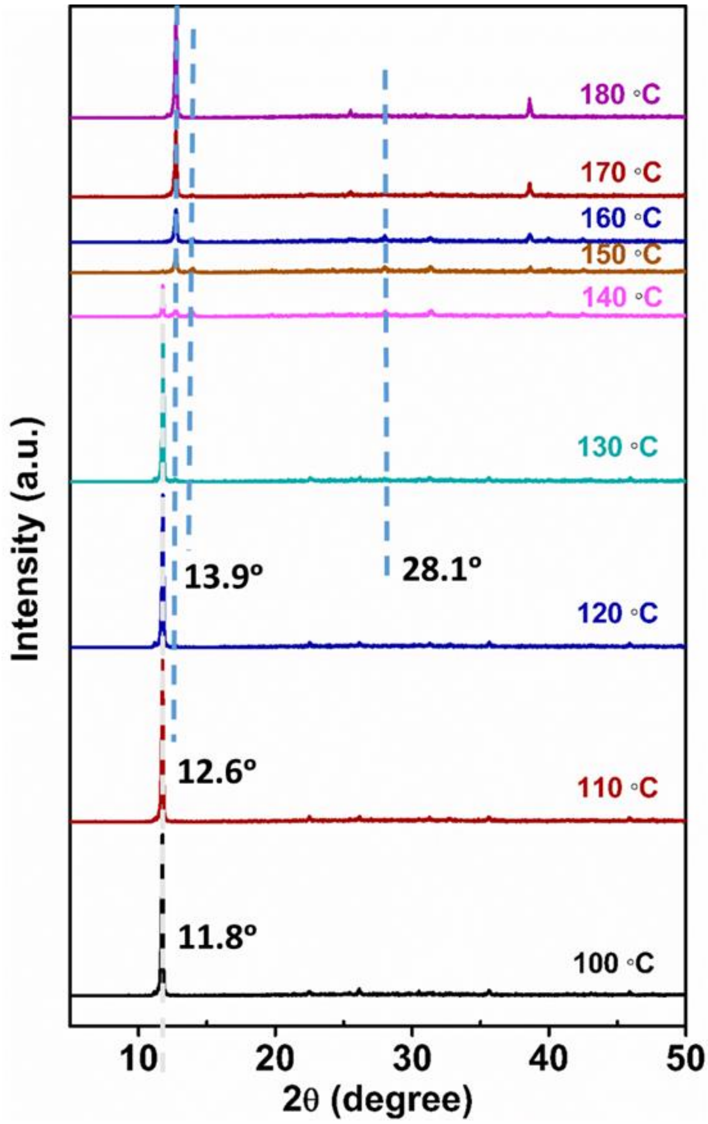


**Figure S1.** In situ X-ray diffraction of the C-$FAPbI_3$ (fabricated via conventional method) films in vacuum. The temperature increases at 5 °C/min, and the soaking time of each scan is 10 min with a characterization speed of 10 °/min.

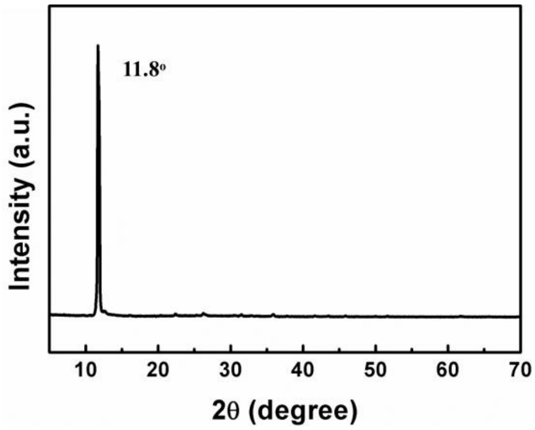


**Figure S2.** The XRD pattern spectrum of δ-$FAPbI_3$ films.

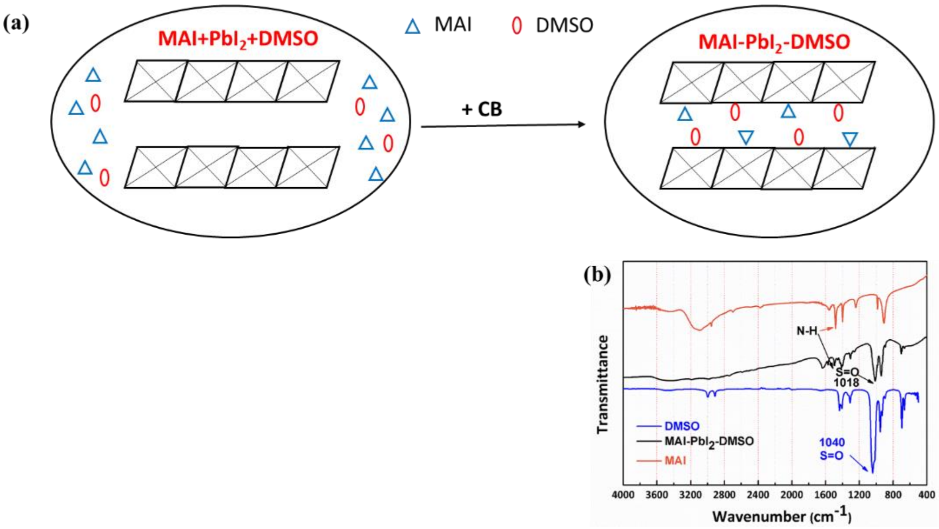


**Figure S3. (**a) Schematic illustration of the template-assisted perovskite mechanism; (b) FTIR spectrums of DMSO, $MAI\text{-}PbI_2\text{-}DMSO$ and MAI.

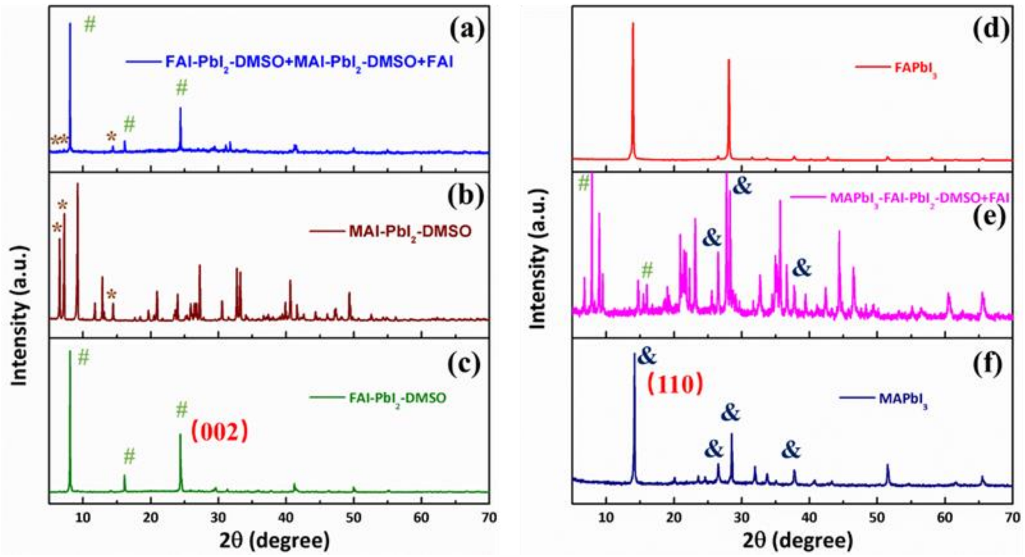

**Figure S4.** XRD patterns of the powders derived from the different intermediates. (a) MAI-$PbI_2$-DMSO+FAI-$PbI_2$-DMSO+FAI, (b) MAI-$PbI_2$-DMSO, (c) FAI-$PbI_2$-DMSO, (d) $FAPbI_3$, (e) $MAPbI_3$-FAI-$PbI_2$-DMSO and (f) $MAPbI_3$.

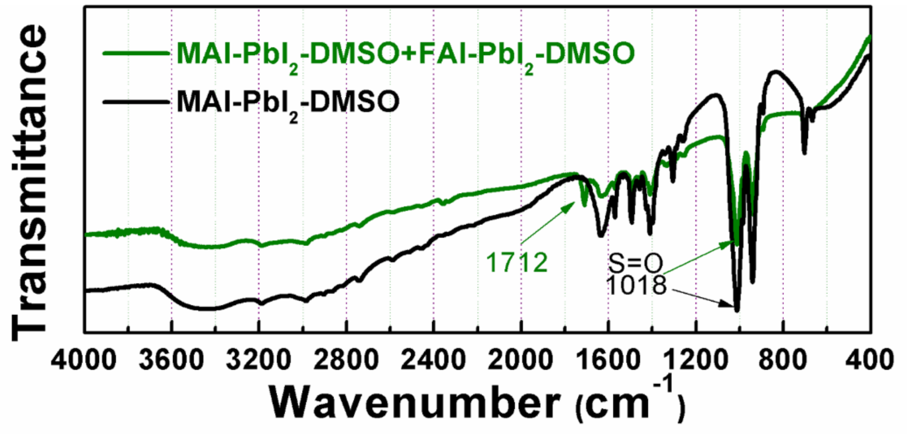


**Figure S5.** FTIR spectrums of the intermediates involving two different precursor combinations.

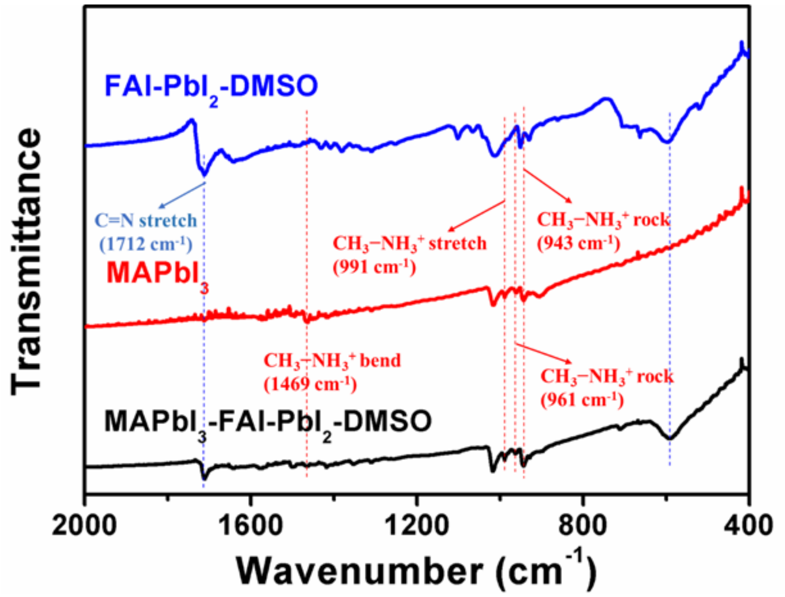


**Figure S6.** FTIR spectrums for the intermediates of FAI-$PbI_2$-DMSO, $MAPbI_3$ and $MAPbI_3$-FAI-$PbI_2$-DMSO.

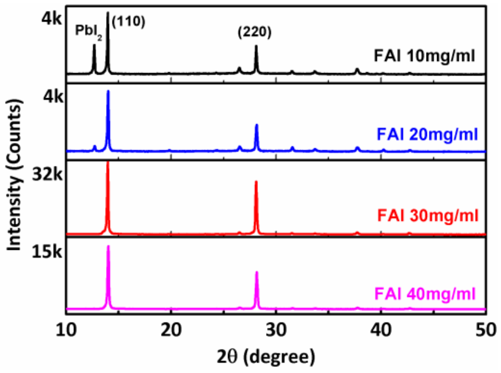


**Figure S7.** XRD patterns of the post-processed films with different FAI concentration.

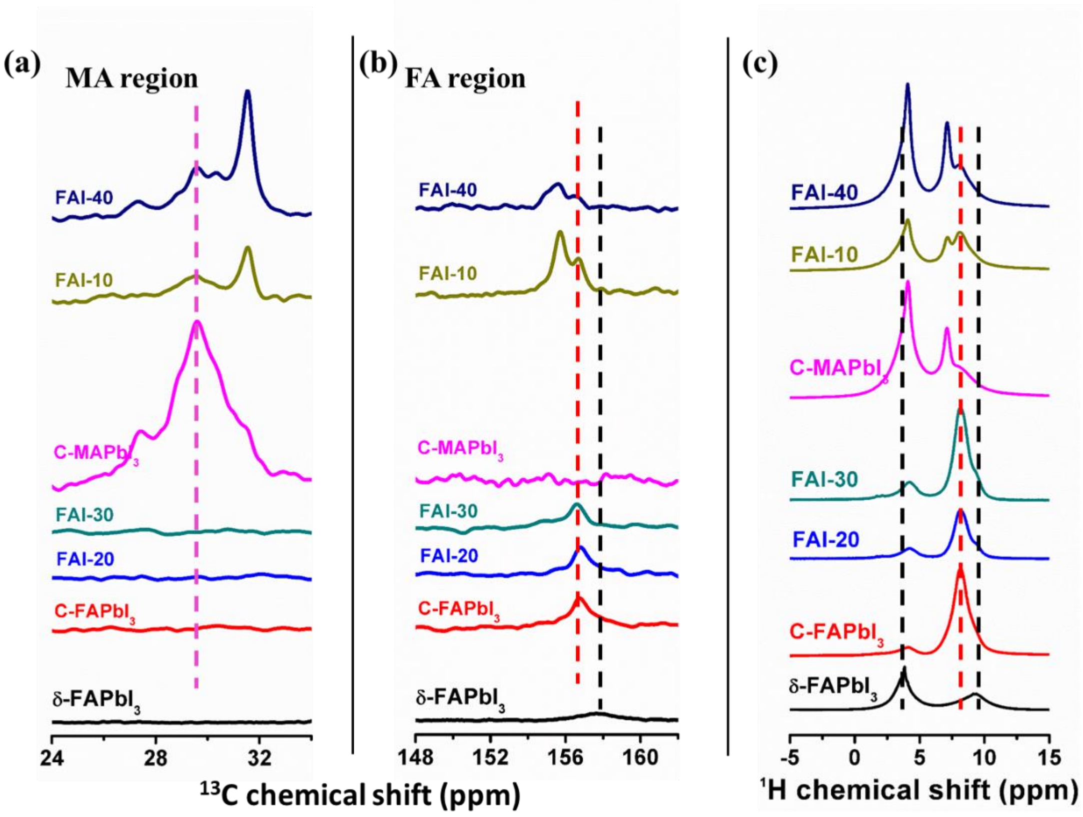


**Figure S8.** Low-temperature (100 ± 3 K) $^{13}C$ CP MAS spectrums of C-FAPbI$_3$, FAI-10, FAI-20, FAI-30, FAI-40 and C-MAPbI$_3$. (a) The MA region of $^{13}C$; (b) the FA region of $^{13}C$. (c) The corresponding $^{1}H$ spectrums.

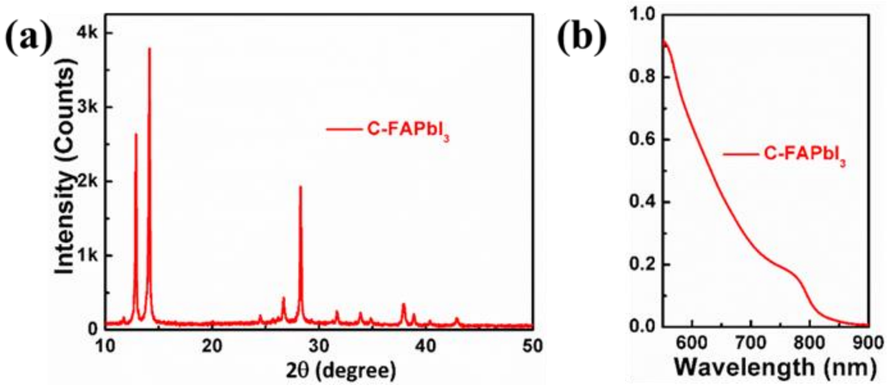


**Figure S9.** (a) XRD pattern of C-$FAPbI_3$; (b) UV-visible spectrum of C-$FAPbI_3$.

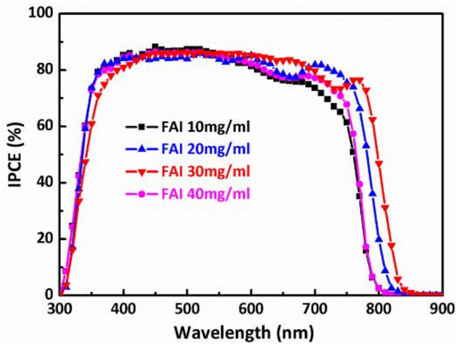


**Figure S10.** External quantum efficiency (EQE) spectrums of perovskite devices based on the films post-processed with different FAI concentration.

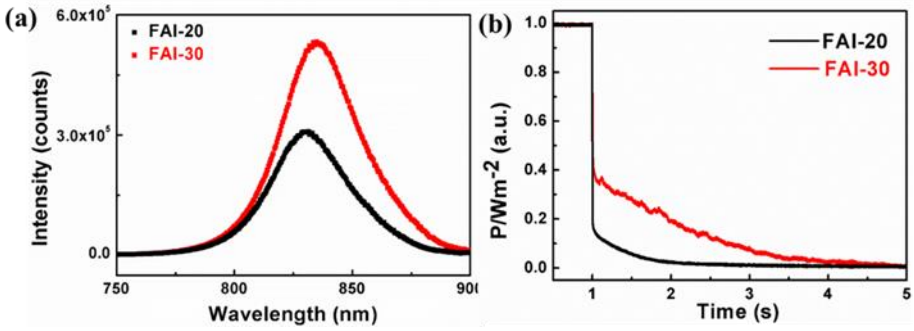


**Figure S11.** (a) Steady-state photoluminescence (PL) spectra and (b) Open-circuit voltage decay (OCVD) measurement.

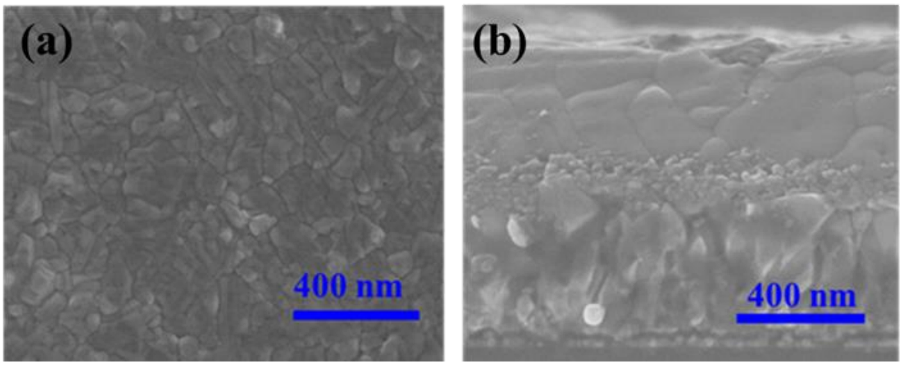


**Figure S12.** (a) Top-view SEM image and (b) cross-sectional SEM of C-$FAPbI_3$ film.

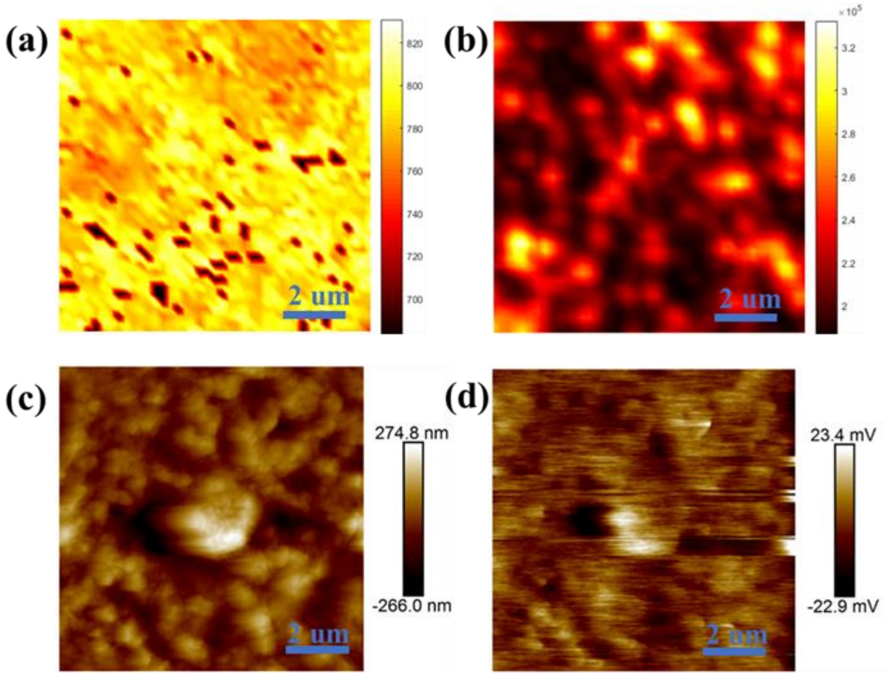


**Figure S13.** (a) Confocal photoluminescence (PL) peak maps, and (b) the corresponding peak intensity maps of C-$FAPbI_3$. (c) Atomic force microscope (AFM) image of C-$FAPbI_3$. (d) Kelvin probe force microscope (KPFM) image of C-$FAPbI_3$. All of the area is $10 \times 10\ \mu m^2$ each.

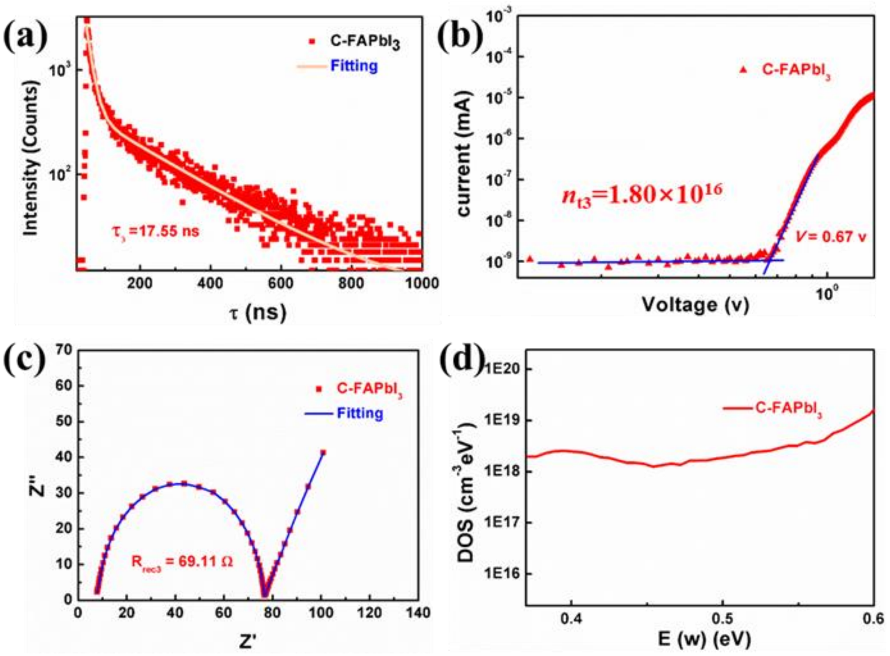


**Figure S14.** Analysis of defects on the C-$FAPbI_3$ films via (a) time-resolved photoluminescence (TRPL), (b) space-charge-limited current (SCLC), (c) electrochemical impedance spectroscopy (EIS) and (d) density of defects measurement.

The TRPL spectrum demonstrated a substantial carrier-hole recombination in the perovskite bulks (Figure S14a), and the SCLC showed a much higher cut-off voltage (0.78 V, Figure S14b), which revealed massive trap-states at the interface. Meanwhile, the EIS showed a lower recombination resistance that stated weak resisting ability for the recombination losses. The spectrum of DOS in Figure S14d presented a higher magnitude order of C-$FAPbI_3$. In summary, for C-$FAPbI_3$, it was hard to reduce trap-states in the perovskite films.

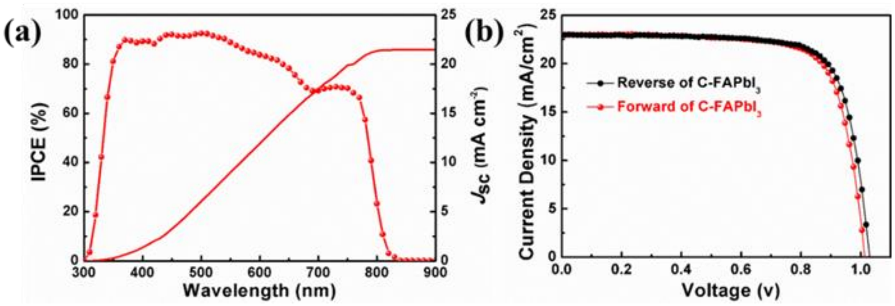


**Figure S15.** (a) EQE spectrum and corresponding integrated current of C-$FAPbI_3$. (b) The corresponding *J*-*V* curves of C-$FAPbI_3$.

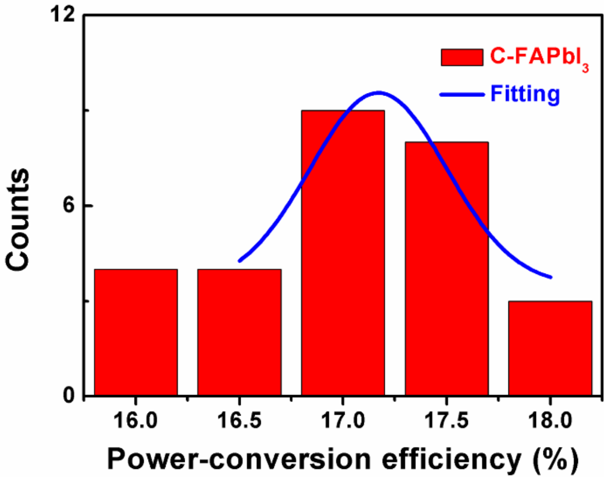


**Figure S16.** Histogram of average efficiencies of C-$FAPbI_3$-based devices (28 devices).

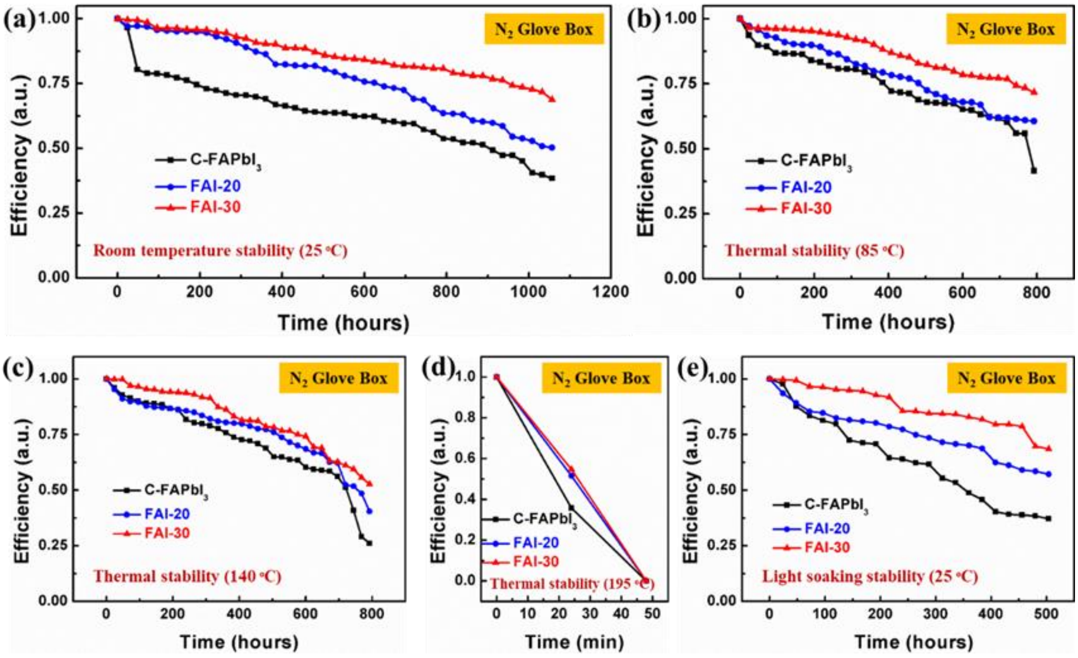


**Figure S17.** Stability data of C-$FAPbI_3$, FAI-20 and FAI-30 based PSCs in nitrogen glove box. (a) Room temperature stability (25 °C); (b) Thermal stability aged at 85 °C; (c) Thermal stability aged at 140 °C; (d) Thermal stability aged at 195 °C; (e) Light soaking stability aged under continuous AM 1.5 light soaking (25 °C).

When aged at room temperature (25 °C), the efficiency decreased to ~75% after 1056 h, which was state-of-art. When aged at 85 °C, the efficiency decreased to ~75% after 800 h, while, it reduced to ~50% when aged at 140 °C. However, it decayed quickly to 0 when aged at 195 °C. Besides, it reduced to ~70% after 500 h under the light soaking. The C-$FAPbI_3$ showed a poor thermal stability than that of FAI-20/FAI-30 devices because the defects and δ-phase in the films.

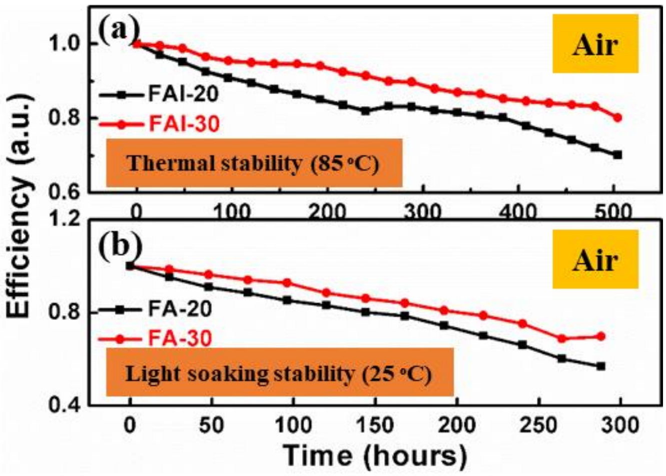


**Figure S18.** (a) Stability data of FAI-30 and FAI-20 based PSC, aged at 85 ºC (the above, at dark), (b) aged under continuous AM 1.5 light soaking (the down, at maximum power point, temperature of ~25 ºC). The stability was measured in the air.

The stability of the final $FAPbI_3$ perovskite devices were tested under thermal stress or light soaking in the laboratory and were shown in Figure S18. The photovoltaic performance was measured every 24 h when aging under 85 ºC and the FAI-30 showed a degradation of 25% after 500 h. We further examined the device light soaking stability of FAI-30 and FAI-20. The FAI-30 based devices showed enhanced light stability in ambient condition by attributing to its high-quality morphology and improved phase stability.

**Table S1.** The fitting results of TRPL for FAI-30, FAI-20 and C-$FAPbI_3$.

| **Samples** | $A_1$ | $\tau_1$ (ns) | $A_2$ | $\tau_2$ (ns) |
|---|---|---|---|---|
| **FAI-30** | 2509.32 | 41.95 | 2744.86 | 173.29 |
| **FAI-20** | 5483.49 | 49.61 | 1174.00 | 130.93 |
| **C-$FAPbI_3$** | 465.10 | 213.29 | 47869.69 | 15.65 |

**Table S2.** The fitting results of EIS for FAI-30, FAI-20 and C-$FAPbI_3$.

| Sample | $R_s$ (Ω) | $R_{rec}$ (Ω) | *CPE-T* | *CPE-P* |
|---|---|---|---|---|
| **FAI-30** | 11.13 | 93.00 | 3.26E-08 | 1.005 |
| **FAI-20** | 10.28 | 72.36 | 4.80E-08 | 0.978 |
| **C-$FAPbI_3$** | 9.21 | 69.11 | 6.31E-08 | 0.925 |

**Table S3.** Performance parameters of FAI-20, FAI-30 and C-$FAPbI_3$ perovskite device. Active area of devices is 0.09 $cm^2$.

| Small devices | Scan direction | $J_{SC}$ ( mA $cm^{-2}$ ) | $J_{SC}$ by EQE ($mA\ cm^{-2}$) | $V_{OC}$ ( V ) | FF ( % ) | PCE(%) |
|---|---|---|---|---|---|---|
| **FAI-30** | Reverse | 24.99 | 24.02 | 1.09 | 78.01 | 21.24 |
| | Forward | 24.96 | | 1.05 | 77.65 | 20.44 |
| **FAI-20** | Reverse | 23.97 | 22.50 | 1.04 | 77.62 | 19.32 |
| | Forward | 23.47 | | 1.02 | 77.00 | 18.52 |
| **C-$FAPbI_3$** | Reverse | 22.62 | 21.45 | 1.029 | 76.04 | 17.95 |
| | Forward | 22.98 | | 1.013 | 75.56 | 17.56 |

## Experimental

## Materials

All the chemicals were commercially available and used as received, including $PbI_2$ (99.99%, Tokyo Chemical Industry, Japan), $CH_3NH_3I$, $HC(NH_2)_2I$ (>99.5%, Xi'an

p-OLED Corp., China), Spiro-OMeTAD (>99.5%, Xi'an p-OLED Corp., China), $TiO_2$ paste (particle size: ~30 nm, crystalline phase: anatase, Dyesol, Australia), DMF, DMSO, chlorobenzene, lithium bis (trifluoromethylsulphonyl) imide (Li-TFSI) and 4-tert-butylpyridine (TBP) (Aldrich, U.S.), chlorobenzene, acetonitrile, methanol, and ethanol were all form Sinopharm Chemical Reagent Co., Ltd.

**Device fabrication**

Devices with theFTO\$TiO_2$\$FAPbI_3$\Spiro-OMeTAD\Au structure is fabricated. FTO glasses were firstly etched with zinc powder and hydrochloric acid (HCl) and then washed by detergent, deionized water, ethanol and acetone The cleaned FTO glasses were placed on a hotplate at the temperature of 500 °C. To deposit $TiO_2$ layer, 10 mL of an acetonitrile/ethanol (with 95:5 volume ratio) solution, nickel acetylacetonate (with 15 mol% magnesium acetatetetrahydrate and 5 mol% lithium acetate, totally 0.02 mol L-1) was sprayed, and $TiO_2$ layer was obtained as a compact layer. After annealing at temperature of 460 °C for 20 min, $TiO_2$ paste (the weight ratio of $TiO_2$ and ethanol is 1:6) was spin-coated on the compact layer at the spin-speed of 5000 rpm as to deposit mesoporous scaffold layer. Later, it was annealed at the temperature of 510°C for 30 min. The perovskite layer is fabricated as mostly reported. Firstly, $PbI_2$/MAI (1:1) was dissolved in 1.2 mol DMSO/DMF (4:1) solution and the mixed solution was dropped on the mesoporous layer and then spun at a speed of 2100 rpm for 10 seconds and then 30 seconds. During the 2$^{nd}$ spinning, chlorobenzene (CB) was dropped. Then, 40 µL solution of FAI dissolved in the IPA solution was dropped on the as prepared film. Subsequently, the as-prepared films were heated at 100 °C for 15

min and 140 °C for 60 min. Finally, the Spiro-OMeTAD was spin-coated on the perovskite layer, after which the gold electrode was thermally evaporated. The conventional method was the two-step sequential deposition process. A thin layer of $PbI_2$ was first deposited on the substrate and formamidinium iodide (FAI) was then applied to the predeposited $PbI_2$ to enable conversion to the perovskite phase, which was then annealled at 150 $^{o}$C.

**Device characterization**

The PCE and *J*–*V* curves were measured with a Keithley 2400 source-meter with a sunlight simulator (XES-300T1, SAN-EI Electric, AM 1.5), which is calibrated using a standard silicon reference cell. The solar cells were masked with a black aperture cover to a define active area of 0.09 $cm^2$. Incident photon to current efficiency (IPCE) was tested as a function of wavelength from 300 nm to 900 nm (Enli Technology), with dual Xenon/quartz halogen light source, measured in DC mode with no bias light. The setup was calibrated with a certified silicon solar cell prior to measurement. Atomic force microscopy (AFM) images were acquired in tapping mode with a 5500 AFM (Agilent Technologies). Time-resolved PL decays were recorded by FLS980 steady-state/transient fluorescence spectrometer (Edinburgh), the pump light wavelength is 485 nm, and the probe light wavelength is 840 nm. Scanning electron microscopy (SEM) images were taken with a SU8010SEM (Hitachi). The in situ XRD characterizations were conducted by a Bruker D8 Advance Davinci powder X-ray diffractometer using a Cu Kα source with a thermal annealing holder. Electrochemical impedance spectroscopy was performed by using the Zahner

electrochemical workstation both in dark and under 1 sun illumination (AM 1.5) conditions at the frequency range of 10 mHz to 1 MHz. The light soaking stability was tested in a solar cell light resistance test system (Model BIR– 50, Bunkoh–Keiki Co., LTD), which equipped with a Class AAA solar simulator; <420 nm UV light was cut off with an optical filter. The thermal stability was tested with controlled temperature at 85 °C in an electronic constant temperature/humidity chamber (THR030FA, Advantec Co., Ltd.). The absorption spectra were recorded using UV/Vis spectrometer (Shimadzu, UV-3600) in the 300 nm ~ 900 nm range. Confocal PL mapping was carried out with a laser confocal Raman spectrometer (Princeton Instruments, Acton Standard Series SP-2558) and a 485 nm laser (PicoQuant LDH-P-C-485, 0.4 mW with a 1% optical density filter), using a home-built confocal microscope on a 10 × 10 $\mu m^2$ sample area. The light intensity dependence measurements and transient photo-voltage decay measurements were performed on an electrochemical workstation (Zahner). A white LED with an intensity of 1000W $m^{-2}$ was used as the light source in the experiments, devices were soaked in light for 2s before the LED light was turned off.
